# RhoGDI2 promotes epithelial-mesenchymal transition via induction of Snail in gastric cancer cells

**DOI:** 10.18632/oncotarget.1733

**Published:** 2014-02-07

**Authors:** Hee Jun Cho, Sun-Mi Park, In-Kyu Kim, In-Koo Nam, Kyoung Eun Baek, Min-Ju Im, Jong-Min Yoo, Seung-Ho Park, Ki-Jun Ryu, Hyun-Tak Han, Hyo-Jin Kim, Soon-Chan Hong, Kwang Dong Kim, Yunbae Pak, Jae Won Kim, Chang Won Lee, Jiyun Yoo

**Affiliations:** ^1^ Division of Applied Life Science (BK21 plus), Research Institute of Life Sciences, Gyeongsang National University, Jinju, Korea; ^2^ Department of Surgery, School of Medicine, Gyeongsang National University, Jinju, Korea; ^*^ These authors contributed equally to the work

**Keywords:** RhoGDI2, EMT, Snail, gastric cancer, invasion

## Abstract

Rho GDP dissociation inhibitor 2 (RhoGDI2) expression correlates with tumor growth, metastasis, and chemoresistance in gastric cancer. Here, we show that RhoGDI2 functions in the epithelial-mesenchymal transition (EMT), which is responsible for invasiveness during tumor progression. This tumorigenic activity is associated with repression of E-cadherin by RhoGDI2 via upregulation of Snail. Overexpression of RhoGDI2 induced phenotypic changes consistent with EMT in gastric cancer cells, including abnormal epithelial cell morphology, fibroblast-like properties, and reduced intercellular adhesion. RhoGDI2 overexpression also resulted in decreased expression of the epithelial markers E-cadherin and β-catenin and increased expression of the mesenchymal markers vimentin and fibronectin. Importantly, RhoGDI2 overexpression also stimulated the expression of Snail, a repressor of E-cadherin and inducer of EMT, but not other family members such as Slug or Twist. RNA interference-mediated knockdown of Snail expression suppressed RhoGDI2-induced EMT and invasion, confirming that the effect was Snail-specific. These results indicate that RhoGDI2 plays a critical role in tumor progression in gastric cancer through induction of EMT. Targeting RhoGDI2 may thus be a useful strategy to inhibit gastric cancer cell invasion and metastasis.

## INTRODUCTION

Though incidence and mortality of gastric cancer have steadily declined in recent decades, it remains the fourth most common type of cancer and the second leading cause of cancer mortality worldwide [1]. Metastasis is still the major cause of mortality in patients with cancer, despite advancements in understanding and treatment of cancer over the last decade. Determining the molecular mechanisms underlying metastatic progression of gastric cancer may provide insights into new therapeutic targets.

Rho GDP dissociation inhibitors (RhoGDIs) were originally identified as negative regulators of Rho GTPases because they bind to a majority of Rho GTPases in the cytoplasm, retaining them in their inactive form and thus preventing interaction with target effector proteins [2, 3]. However, recent reports suggest that RhoGDIs also act as positive regulators of Rho GTPases as they are associated with active forms of Rho, Rac, and Cdc42 [4, 5]. These positive interactions maintain Rho in an active form by inhibiting both intrinsic and GTPase-activating protein-stimulated GTPase activities of Rho GTPases. Unlike other members of the family (such as RhoGDI1 and RhoGDI3), RhoGDI2 is preferentially expressed in hematopoietic cells, and seems to have a narrow selectivity and lower binding affinity for Rho GTPases [6]. A significant role of RhoGDI2 in cancer has previously been identified in several studies. RhoGDI2 expression is inversely correlated with invasive capacity in bladder cancer cell lines [7], and reduced RhoGDI2 expression is associated with poor prognosis patients with advanced bladder cancer [8]. In contrast, RhoGDI2 mRNA expression is significantly higher in ovarian adenocarcinomas than in benign adenomas [9]. Consistent with this finding, RhoGDI2 is overexpressed in human breast cancer cell lines, and it increases cancer cell invasiveness and motility *in vitro* [10]. We have also shown that RhoGDI2 expression is positively correlated with tumor progression and metastatic potential in gastric cancer [11].

Epithelial to mesenchymal transition (EMT) is an essential morphologic conversion that occurs during embryonic development. There is increasing evidence that a similar process occurs during cancer progression, by which tumor cells acquire the capacity to migrate, invade, and metastasize [12]. It has been shown that EMT can be induced by signaling from several growth factor receptors and chemokine receptors [13-15]. Loss of cell-cell adhesion is a prerequisite of EMT and involves functional loss of E-cadherin. The zinc finger transcription factors of the Snail family have been implicated in this repression [16-18]. Currently these important EMT drivers, which have a central role in the biological significance to EMT activation, are shown to correlate significantly with poor clinical prognosis in various types of cancers [19-22].

In this study, we show for the first time involvement of RhoGDI2 in EMT of human gastric cancer cells. We also present evidence suggesting that this tumorigenic activity is associated with the ability of RhoGDI2 to repress E-cadherin via upregulation of Snail expression.

## RESULTS

### RhoGDI2 induces EMT in gastric cancer cells

**Figure 1:**
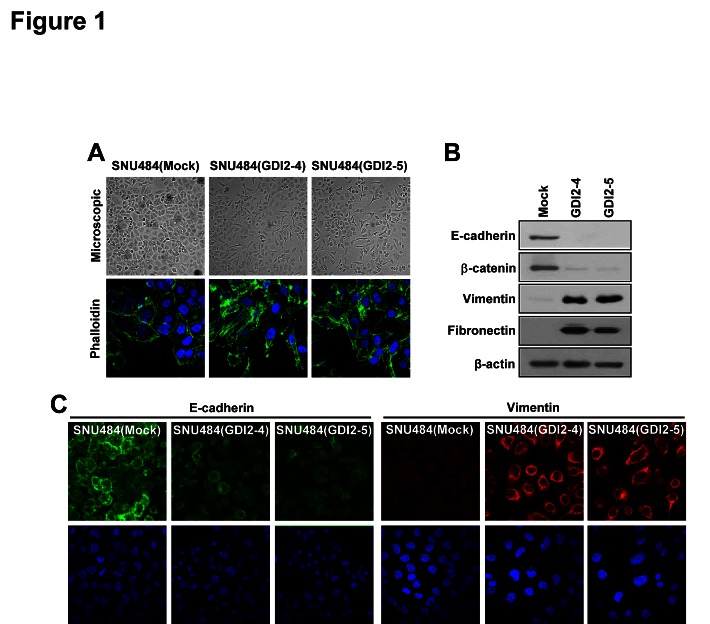
RhoGDI2 induces EMT in gastric cancer cells (A) Representative phase-contrast microscopic images (upper) and fluorescence microscopic staining images of Phalloidin (lower) in RhoGDI2-overexpressing SNU-484(GDI2-4 and GDI2-5) cells. (B) Representative immunoblot for epithelial markers, E-cadherin and β-catenin, and mesenchymal markers, Vimentin and Fibronectin, in RhoGDI2-overexpressing SNU-484(GDI2-4 and GDI2-5) cells. (C) Representative fluorescence microscopic staining images of E-cadherin and Vimentin in RhoGDI2-overexpressing SNU-484(GDI2-4 and GDI2-5) cells.

RhoGDI2 promotes tumor growth and malignant progression in gastric cancer [11], and induction of EMT is associated with tumor progression and poor prognosis in gastric cancer [23]. We therefore examined whether RhoGDI2 may be involved in the control of EMT in gastric cancer cells using SNU-484 cells in which RhoGDI2, which is not normally expressed, was ectopically overexpressed and have increased invasive and metastatic ability [11]. Interestingly, the morphology of RhoGDI2-overexpressing SNU-484(GDI2-4 and GDI2-5) cells was distinct from that of control (Mock) cells. While control cells remained tightly attached with typical epithelial cell characteristics, RhoGDI2-overexpressing SNU-484 cells were more spread out, and lost their cell-cell contacts (Fig. 1A, upper). Confocal microscopy of phalloidin-stained cells also confirmed the presence of filopodia, lammelopodia, and microspikes in RhoGDI2-overexpressing SNU-484 cells, while control cells showed less staining with no cellular outgrowth (Fig. 1A, lower).

To investigate the mechanism underlying the morphological changes, we examined the expression of epithelial markers (E-cadherin and β-catenin) and mesenchymal markers (vimentin and fibronectin). Protein levels of epithelial markers were significantly decreased, whereas levels of mesenchymal markers were significantly increased in RhoGDI2-overexpressing SNU-484 cells compared with control cells (Fig. 1B). Immunofluorescence analysis also revealed less E-cadherin staining in the cell membrane region and a higher level of vimentin staining in the cytoplasmic region in RhoGDI2-overexpressing SNU-484 cells (Fig. 1C). These results suggest that RhoGDI2 acts as a positive regulator of EMT in gastric cancer cells.

### RhoGDI2 induces Snail expression

**Figure 2:**
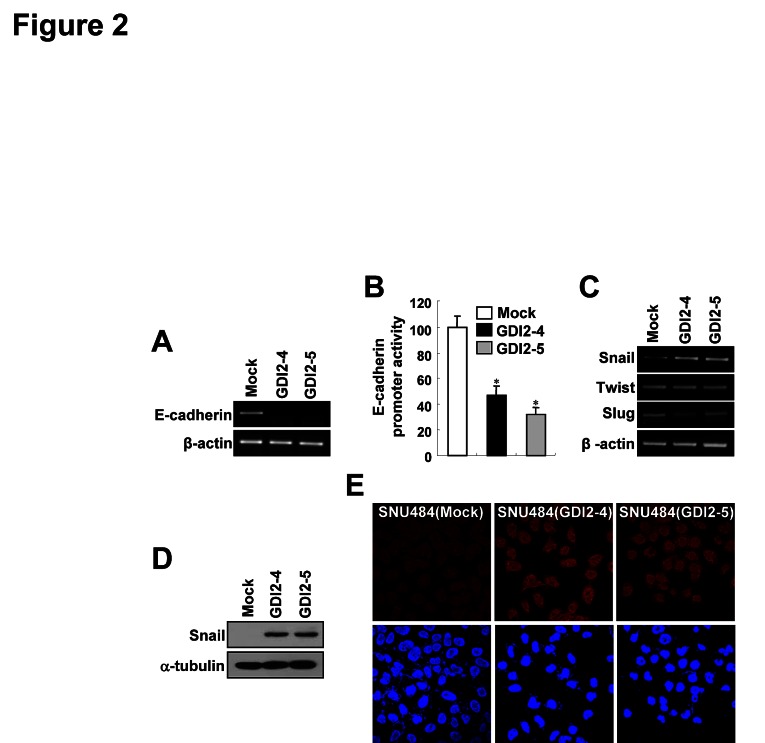
RhoGDI2 increases Snail expression mRNA expression (A) and promoter activity (B) of E-cadherin in RhoGDI2-overexpressing SNU-484(GDI2-4 and GDI2-5) cells. Data are mean ± SD of three individual experiments, each in triplicate. , P < 0.01 as determined by paired Student t test. (C) mRNA expression of Snail, Twist, and Slug in RhoGDI2-overexpressing SNU-484(GDI2-4 and GDI2-5) cells. (D) Representative immunoblot for Snail in RhoGDI2-overexpressing SNU-484(GDI2-4 and GDI2-5) cells. (E) Representative fluorescence microscopic staining images of Snail in RhoGDI2-overexpressing SNU-484(GDI2-4 and GDI2-5) cells.

A hallmark of EMT is the loss of E-cadherin. Transcription of the E-cadherin gene is silenced in various carcinomas, and it is thought to be a tumor suppressor [12]. mRNA expression and promoter activity of the E-cadherin gene are markedly repressed in RhoGDI2-overexpressing SNU-484 (GDI2-4 and GDI2-5) cells compared with control (Mock) cells (Fig. 2A and B). The zinc finger-containing proteins Snail and Slug and the helix-loop-helix transcription factor Twist repress E-cadherin expression and induce EMT in gastric cancer [24-26]. We thus assessed the expression levels of the above E-cadherin regulators in RhoGDI2-overexpressing SNU-484 cells to determine the effect of RhoGDI2. We found no differences in the expression of Slug and Twist (Fig. 2C), but Snail mRNA and protein expression was increased in RhoGDI2-overexpressing SNU-484 cells (Fig. 2C and D). We also found that transient expression of RhoGDI2 in HEK293T cells increases Snail and decreases E-cadherin, as well as decreasing promoter activity of the E-cadherin gene (Supplementary Fig. 1), suggesting that Snail is a direct target of RhoGDI2. Furthermore, we found that the increased Snail protein is localized in the nucleus of RhoGDI2-overexpressing SNU-484 cells (Fig. 2E). These results suggest that Snail is positively regulated by RhoGDI2 and that Snail might play an important role in RhoGDI2-induced gastric cancer cell EMT.

### Snail is essential for RhoGDI2-induced EMT in gastric cancer cells

**Figure 3:**
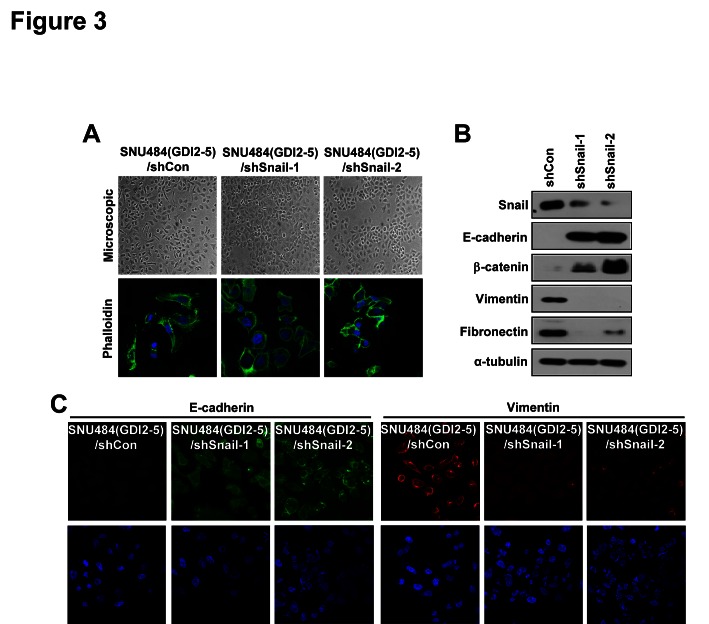
Snail is critical for RhoGDI2-induced EMT in gastric cancer cells (A) Representative phase-contrast microscopic images (upper) and fluorescence microscopic staining images of Phalloidin (lower) in Snail-depleted RhoGDI2-overexpressing SNU-484(GDI2-5) cells (shSnail-1 and shSnail-2). (B) Representative immunoblot for epithelial markers, E-cadherin and β-catenin, and mesenchymal markers, Vimentin and Fibronectin, in Snail-depleted RhoGDI2-overexpressing SNU-484(GDI2-5) cells (shSnail-1 and shSnail-2). (C) Representative fluorescence microscopic staining images of E-cadherin and Vimentin in Snail-depleted RhoGDI2-overexpressing SNU-484(GDI2-5) cells (shSnail-1 and shSnail-2).

To investigate whether Snail is an important mediator for RhoGDI2-induced EMT in gastric cancer cells, expression of Snail was downregulated in RhoGDI2-overexpressing SNU-484(GDI2-5) cells by lentiviral shRNA (shSnail-1 and shSnail-2). Depletion of Snail expression resulted in reversal of the EMT-associated morphological changes induced by ectopic expression of RhoGDI2. Snail-depleted RhoGDI2-overexpressing SNU-484(GDI2-5) cells (shSnail-1 and shSnail-2) were tightly attached, showing typical epithelial cell characteristics, with no cellular outgrowth (Fig. 3A). Repression of epithelial markers (E-cadherin and β-catenin) and enhanced expression of mesenchymal markers (vimentin and fibronectin) in RhoGDI2-overexpressing SNU-484(GDI2-5) cells was rescued through the knockdown of Snail expression (Fig. 3B and C). The above results suggest that upregulation of Snail expression is critical for the induction of EMT in RhoGDI2-overexpressing gastric cancer cells.

### Downregulation of Snail suppresses RhoGDI2-induced gastric cancer cell invasion and migration

**Figure 4:**
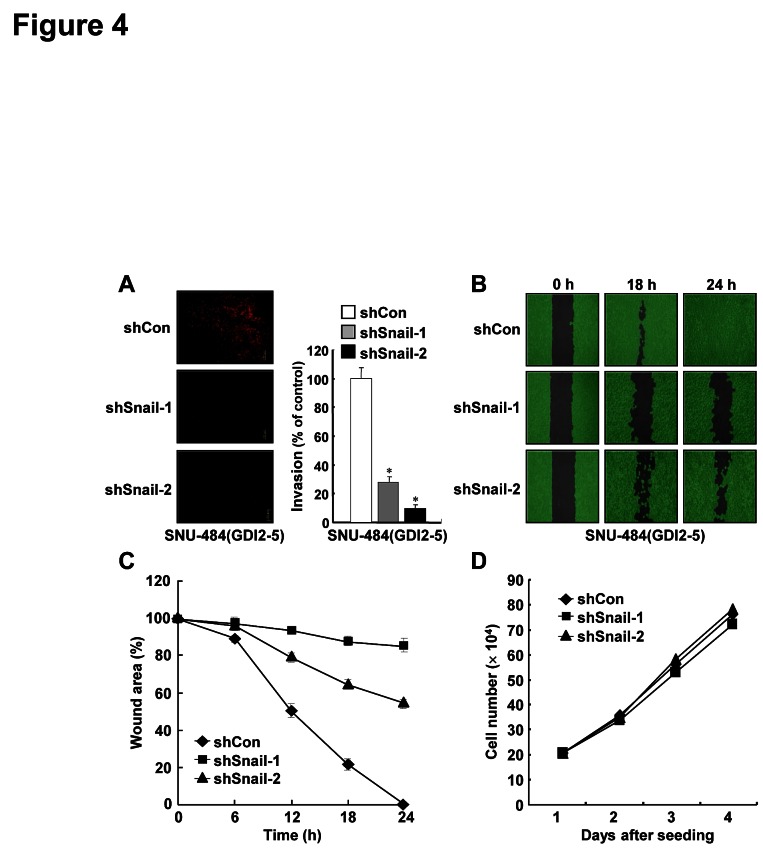
Inhibition of Snail suppresses RhoGDI2-induced gastric cancer cell invasion and migration (A) Representative images of invading cells through the matrigel-coated membrane stained by propidium iodide. Quantitative data of invasion assay are expressed relative to the invasion ability of SNU-484(GDI2-5/shCon) cells. Data are mean ± SD of three individual experiments, each in triplicate. , *P* < 0.01 as determined by paired Student *t* test. (B) Representative images of migrating cells obtained at indicated time points after wound formation by phase contrast microscopy and using Wimasis Image Analysis software. (C) Quantitative analysis of wound healing assay by using the WimScratch software (Wimasis). Data represent the percentage of wound area at indicated time points in control and Snail-depleted SNU-484(GDI2-5) cells. (D) Effect of Snail depletion on the proliferation of RhoGDI2-overexpressing SNU-484(GDI2-5) cells.

Since RhoGDI2 promotes gastric cancer cell invasion [11], we examined whether depletion of Snail expression could alter the migration and invasive properties of RhoGDI2-overexpressing gastric cancer cells. Downregulation of Snail expression considerably decreased the invasiveness of RhoGDI2-overexpressing SNU-484(GDI2-5) cells (shSnail-1 and shSnail-2) compared with control (shCon) cells (Fig. 4A). Next, we checked the migration ability of Snail-depleted SNU-484(GDI2-5) cells by using a wound healing assay. For this analysis, we used a culture-insert, where a non-bias cell-free gap is produced as the “wound” when the culture-insert is removed. Immediately after removal of the culture-insert, cell images were obtained at various time points (0-30 h) under a light microscope. After 24 h, complete wound closure (100%) was achieved in RhoGDI2-overexpressing SNU-484(GDI2-5) control (shCon) cells, whereas only 11% (shSnail-1) and 47% (shSnail-2) wound closure was achieved in Snail-depleted SNU-484(GDI2-5) cells (Fig. 4B and C). Taken together, these results suggest that upregulation of Snail expression plays a key role in RhoGDI2-induced gastric cancer cell migration and invasion. To exclude the possibility that the effect of Snail on migration and invasion of RhoGDI2-overexpressing gastric cancer cells was attributable to different proliferation rates, we compared growth rates of Snail-depleted SNU-484(GDI2-5) cells (shSnail-1 and shSnail-2) with those of control (shCon) cells. Under identical growth conditions, all cells exhibited similar growth rates (Fig. 4D), indicating that the decreased migration and invasion via depletion of Snail expression was not associated with proliferation rate.

### RhoGDI2 increases Snail expression through Rac1-mediated NF-kB activation

**Figure 5:**
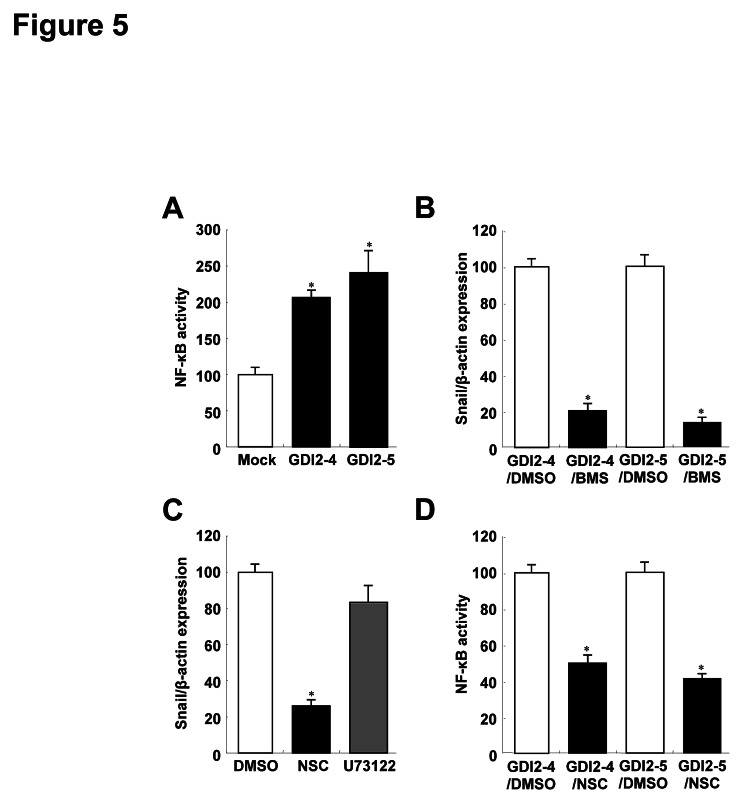
RhoGDI2 increases Snail expression through Rac1-mediated NF-κB activation (A) NF-κB activities of RhoGDI2-overexpressing SNU-484(GDI2-4 and GDI2-5) cells. (B) Effect of BMS-345541 (BMS) on Snail expression. (C) Effect of NSC23766 (NSC) and U73122 on Snail expression. (D) Effect of NSC23766 on NF-κB activation of RhoGDI2-overexpressing SNU-484(GDI2-4 and GDI2-5) cells. Data are mean ± SD of three individual experiments, each in triplicate. , *P* < 0.01 as determined by paired Student *t* test.

NF-κB is directly involved in transcriptional regulation of Snail expression [27-29], and thus we examined the involvement of NF-κB in RhoGDI2-induced Snail expression in gastric cancer cells. We found that NF-κB activity was significantly higher in RhoGDI2-overexpressing cells than that in control cells (Fig. 5A), and that suppression of NF-κB activity by IKK inhibitor III (BMS-345541) significantly reduced Snail expression in RhoGDI2-overexpressing SNU-484(GDI2-4 and GDI2-5) cells (Fig. 5B). These results suggest that NF-κB activation is important for RhoGDI2-induced Snail upregulation in gastric cancer cells.

RhoGDI2 is a critical regulator of Rac1 in many cancer cells [30, 31] and PLCγ is activated by RhoGDI2 expression in gastric cancer cells [32]. We therefore examined whether suppression of Rac1 or PLC activity affects Snail expression in RhoGDI2-overexpressing gastric cancer cells. Suppression of Rac1 activity by NSC23766 significantly inhibited Snail expression in RhoGDI2-overexpressing SNU-484(GDI2-4 and GDI2-5) cells (Fig. 5C); however, PLC inhibitor U73122 had no effect on Snail expression. NF-κB activity was markedly suppressed by inhibition of Rac1 activity (Fig. 5D). Taken together, these results suggest that RhoGDI2-mediated Rac1 activation is important for NF-κB activation, which induces Snail upregulation in gastric cancer cells.

### Snail is not required for RhoGDI2-induced chemoresistance in gastric cancer cells

**Figure 6:**
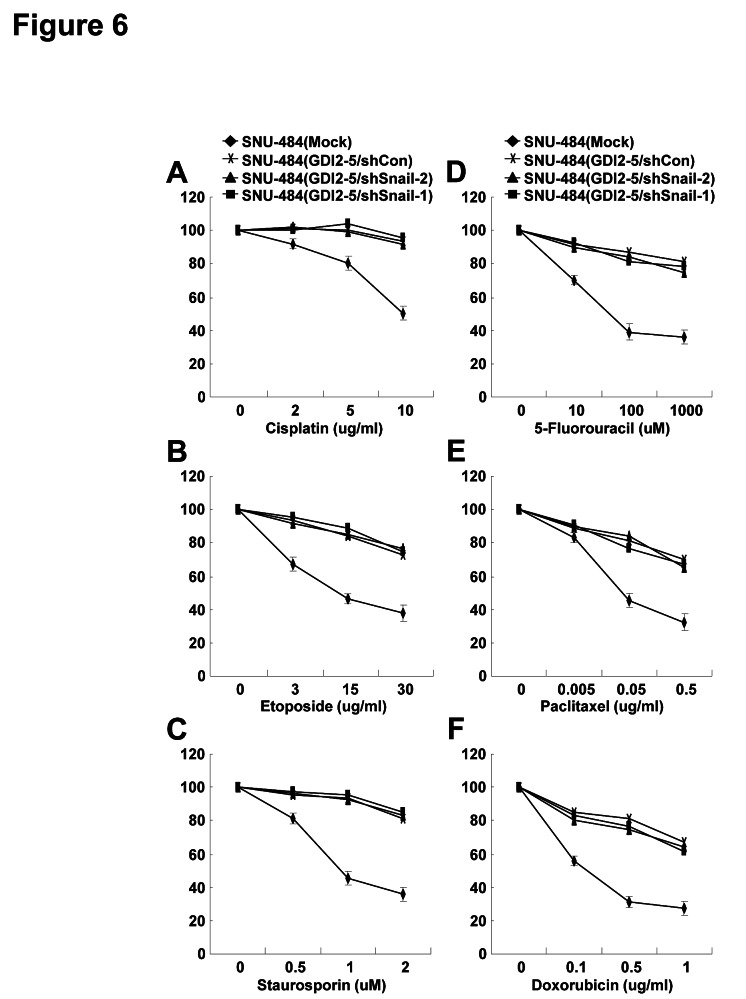
Snail is not required for RhoGDI2-induced chemoresistance in gastric cancer cells Viability of Snail-depleted RhoGDI2-overexpressing SNU-484(GDI2-5) cells (shSnail-1 and shSnail-2) after treatment with the indicated concentrations of cisplatin (A), etoposide (B), staurosporin (C), 5-fluorouracil (D), paclitaxel (E), or doxorubicin (F) for 24 h. Data are mean ± SD of three individual experiments, each in triplicate.

We previously showed that RhoGDI2 protects gastric cancer cells against apoptosis induced by various chemotherapeutic agents [33-35], as well as promotes gastric cancer cell invasion. Furthermore, Snail mediates chemoresistance in many cancer cell types [36-39]. To determine whether upregulation of Snail expression is required for RhoGDI2-induced chemoresistance in gastric cancer cells, we used various chemotherapeutic drugs such as cisplatin, etoposide, staurosporin, 5-fluorouracil, paclitaxel, and doxorubicin to treat Snail-depleted RhoGDI2-overexpressing gastric cancer cells. Consistent with our previous results, overexpression of RhoGDI2 resulted in increased cell viability compared with control cells (Fig. 6) [33]; however, depletion of Snail expression did not change viability of RhoGDI2-overexpressing gastric cancer cells in response to chemotherapeutic drugs (Fig. 6). These results indicate that upregulation of Snail expression does not contribute to RhoGDI2-induced chemoresistance in gastric cancer cells.

## DISCUSSION

EMT is a critical cellular mechanism during tumor progression and metastasis development and results in enhanced cell motility and invasion. However, few studies have examined the possible role of EMT in gastric cancer [23-26]. Here, we show that RhoGDI2, which is frequently overexpressed in advanced stage gastric cancer, is an important mediator of EMT. Furthermore, we demonstrated the mechanism by which RhoGDI2 increases Snail expression. RhoGDI2 activates NF-κB in a Rac1-dependent manner; activated NF-κB is critical for Snail upregulation in RhoGDI2-overexpressing gastric cancer cells, indicating the central role of RhoGDI2 in regulating Snail expression and consequently EMT.

Accumulating evidence suggests that RhoGDI2 is differentially expressed in human cancers [6]. For example, RhoGDI2 is selectively downregulated in Hodgkin lymphoma cells when compared with non-Hodgkin lymphoma cells, and this loss of RhoGDI2 expression may contribute to the resistance to apoptosis in Hodgkin lymphoma cells [40]. Similarly, Theodorescu and colleagues have shown the expression of RhoGDI2 in the T24 human bladder cancer cell line, but not in the more aggressive T24T lineage, and that forced expression of RhoGDI2 in highly aggressive T24T cells suppresses cancer cell invasion, motility, and lung metastasis [41, 7]. Together these data implicate RhoGDI2 as a metastasis suppressor in bladder cancer. However, other lines of research have suggested that RhoGDI2 is also associated with advanced stage tumors and increased metastatic potential of tumor cells in ovarian and breast cancers [9, 10]. Our previous work suggests that increased RhoGDI2 expression is associated with advanced stage gastric tumors and lymph node metastasis [11].

While there are many reports implicating RhoGDI2 in tumor growth and malignant progression in numerous cancer types, the exact mechanism of RhoGDI2 in tumor growth and malignant progression is poorly understood. To identify possible mechanisms for RhoGDI2-induced invasion and migration in gastric cancer cells, we examined the effects of RhoGDI2 overexpression on EMT. EMT, as evidenced by drastic changes in cell morphology and reversal of the expression of epithelial markers (E-cadherin and β-catenin) and mesenchymal markers (vimentin and fibronectin), was activated in RhoGDI2-overexpressing gastric cancer cells. RhoGDI2 is known to be increased in gastric cancer cell invasion and migration, but it has not been determined whether RhoGDI2 overexpression results in EMT in gastric cancer cells. This is the first report, to our knowledge, suggesting that RhoGDI2 can induce EMT.

In our study, RhoGDI2-induced EMT was associated with increased Snail gene expression, known to be a main contributor to E-cadherin suppression. A series of recent reports showed that NF-κB is directly involved in the transcriptional regulation of Snail expression [27-29], and NF-κB activation is also involved in EMT [42-44]. Overexpression of the active subunit of NF-κB, p65, is sufficient for induction of EMT [42] and NF-κB signaling is required for transcriptional activation of the Snail gene by GSK-3 inhibition [45]. Similarly, Kim et al. showed that Snail expression is regulated by NF-κB during EMT in MCF10A cells overexpressing insulin-like growth factor receptors [46]. In our study, we investigated a potential role for NF-κB in Snail gene expression. Basal NF-κB activity was significantly increased in RhoGDI2-overexpressing gastric cancer cells and inhibition of NF-κB activity resulted in marked reduction of Snail expression. Therefore, RhoGDI2-induced EMT may result from NF-κB-mediated Snail expression in gastric cancer cells.

RhoGDIs are generally thought to be negative regulators of Rho GTPases, which act through binding and retaining Rho GTPases in inactive, cytosolic forms that are unable to interact with downstream target molecules. Zhang et al. recently showed that RhoGDI2 associates with Rac1 in MDA-MB-231 human breast cancer cells, and RhoGDI2 depletion results in constitutive Rac1 activation [30]. However, several lines of evidence, including ours, indicate that RhoGDI2 may act as a positive regulator of Rac1. For example, Moissoglu et al. recently described RhoGDI2 as an activator of Rac1 in T24 and UMUC3 human bladder cancer cells [31]. Huang et al. also showed that RhoGDI2 induces hypertrophic growth and cell migration through Rac1 activation in H9c2 cardiomyoblast cells [47], and interestingly, showed that RhoGDI2 overexpression enhances Rac1 expression, leading to increased levels of membrane-associated Rac1. We also found that Rac1 is positively regulated by RhoGDI2 in gastric cancer cells (data not shown) and activated Rac1 mediates NF-κB activation, which induces Snail upregulation in RhoGDI2-overexpressing gastric cancer cells. Numerous studies suggest that Rac1 regulates intracellular ROS production via increased NADPH oxidase activity, leading to NF-κB activation [48-50]. Therefore, we are now examining whether Rac1-dependent redox signals are involved in RhoGDI2-mediated NF-κB activation and consequent Snail expression.

Overall, the present study shows that RhoGDI2 overexpression is involved in acquisition of the mesenchymal phenotype of gastric cancer cells, and that Rac1-dependent NF-κB activation and subsequent Snail expression is essential for induction of EMT.

## MATERIALS AND METHODS

### cellcultures and reagents

Human gastric cancer cell lines SNU-484 derived RhoGDI2-overexpressing cells (GDI2-4 and GDI2-7) were maintained in RPMI-1640 medium (Sigma). Human embryo kidney cell line HEK293T cells were purchased from American Type Culture Collection (ATCC) and cultivated in DMEM (Sigma). All cell lines were maintained as mono-layer cultures in each optimal medium supplemented with 10% heat-inactivated FBS (Gibco, Invitrogen) and 2% of a penicillin-streptomycin (antibiotic-antimycotic) mixture (Gibco, Invitrogen) The SNU-484 cells stably transfected with RhoGDI2 were described in our previous report [11]. BMS-345541, NSC23766, and U73122 were purchased from Calbiochem. Cisplatin, etoposide, staurosporin, 5-fluorouracil, paclitaxel and doxorubicin were purchased from Sigma.

### Antibodies and western blot analysis

Mouse anti-E-cadherin, anti-β-catenin, and anti-Fibronectin antibodies were purchased from BD Transduction Laboratories. Mouse anti-Vimentin antibody was purchased from Santa Cruz Biotechnology. Rabbit anti-E-cadherin and Mouse anti-Snail antibodies were purchased from Cell Signaling Technology. Mouse anti-α-tubulin antibody was purchased from Sigma. For western blot analysis, cells were harvested after defined time and lysed in lysis buffer (20 Mm Tris (pH 7.4), 2 mM EDTA, 150 mM sodium chloride, 1 mM sodium deoxycholate, 1% Triton X-100, 10% glycerol, 2 pills protease inhibitor cocktail (Roche)) on ice for 1 h and centrifuged at 13,000 rpm for 15 min. Cell lysates were separated by 8–12% SDS-PAGE and transferred to a polyvinylidene difluoride membrane (Amersham Bioscience). Subsequently, the membrane was incubated in TBST supplemented with 5% non-fat dry milk and probed with the appropriate primary antibodies. The bound antibodies were visualized with a suitable secondary antibody conjugated with horseradish peroxidase using enhanced chemiluminescence (ECL) reagent WESTSAVE up (AbFRONTIER, Korea).

### Immunofluorescence analysis

Cells were seeded on glass coverslips at a density of 1 × 10^5^ cells/12 well plate. After 48 h, cells were rinsed three times with PBS, and fixed for 10 min at room temperature in 3.7% paraformaldehyde fixative. Fixed cells were permeabilized with PBS containing 0.1% Triton X-100 for 20 min, washed three times in PBS and blocked with blocking solution (1% BSA in PBS) for 1 h at room temperature. After blocking, cell were incubated with primary anti-Snail, anti-Vimentin, anti-β-catenin (mouse, 1:100 dilution) and anti-E-cadherin (Rabbit, 1:100 dilution) antibodies in 1% BSA for overnight at 4^o^C. Following three washes with PBS, cells were incubated with Alexa Fluor 594 labeling goat anti-mouse IgG (Cambrex), Fluorescein isothiocyanate (FITC) labeling anti-mouse, anti-rabbit IgG and FITC conjugated-Phalloidin (Sigma) for 1 h at room temperature. Labeled cells were rinsed three times with PBS, mounted in Fluorescent Mounting Medium. The cells were examined by confocal laser-scanning microscope (FV-1000, Olympus). Acquired images were manipulated with FV10-ASW 1.7 Viewer (Olympus).

### Reverse Transcription-PCR analysis

Total RNA was isolated using an RNeasy mini kit (Qiagen) according to the manufacturer's instructions. RT-PCR was performed using a Maxime RT-PCR PreMix kit (Intron, Korea). 200 ng of total RNA and specific primer were added into the Maxime RT-PCR PreMix tubes and RNase-free water was added to a total volume of 20 µl. RT-PCR was performed using a Thermo Electron PCR thermal cycler. Real-time PCR was performed using SSoFast^TM^ EvaGreen Supermix^®^ and CFX96^TM^ Real-time detection system (Bio-Rad Laboratories). The used primers were described in Supplementary Table 1.

### Reporter gene analysis

Promoter activity was determined using a dual-luciferase reporter assay system (Promega). Briefly, the cells (3 × 10^5^ cells/well) were plated in 12-well plates overnight and transiently transfected with the E-cadherin or NF-κB reporter plasmid/pGL3-Basic plasmid using the FuGENE^®^ 6 reagent (Promega). Cells were then incubated in culture medium for 48 h, and firefly and Renilla luciferase activities in the cell lysates were measured using a 1420 Luminescence counter (Perkin Elmer). Relative luciferase activities were calculated by normalizing the promoter-driven firefly luciferase activity to Renilla luciferase activity.

### RNA interference experiments

The short hairpin RNA (shRNA)-expressing lentiviral transduction particles for targeting the Snail gene was constructed by inserting synthetic double-stranded oligonucleotides (shSnail-1; 5'-CCGGCCACTCAGATGTCAAGAAGTACTC GAGTACTTCTTGACATCTGAGTGGTTTTTG-3', shSnail-2; 5'-CCGGCCAGGCTCGAAAGGCCTTCAAC TCGAGTTGAAGGCCTTTCGAGCCTGGTTTTTG-3') into the pLKO.1 lentiviral vector and the Non-Target shRNA Control Transduction Particles were purchased from Sigma. 48 h after transduction, 1 µg/ml puromycin (Clontech) was added to the cultures for selection. After 14 days, puromycin-resistant cell pools were established.

### Invasion and migration assay

The invasion ability of cancer cells was assessed using a matrigel-based transwell system. Briefly, 24-well cell culture plate inserts with 8-μm pore size polycarbonate membrane (Corning) were precoated with 100 μl matrigel/RPMI-1640 solution (2.2 mg/ml, BD Bioscience) and incubated at 37 for 2 h or overnight at 4. All the cells were preincubated in serum-free media with or without inhibitors for 24 h. 2.5 × 10^5^ cells in 250 µl of medium (no serum) were placed in the insert and allowed to invade for 48 h. The lower chamber was filled with 750 µl of appropriated media containing 20% FBS. After incubation, medium remaining on top of the insert were removed by pipetting and non-invading cells on the upper surface of the insert membrane were removed with cotton swab. After washing twice with PBS, the insert membranes were fixed for 10 min with MeOH/Acetic acid (3:1) at -20 and stained with 50 µg/ml propidium iodide (Sigma) for 20 min at 37. The upper surface of the insert membrane was gently scrubbed with cotton swab again and washed with distilled water. Membranes were cut and mount on slide glass and the number of invaded cells was counted microscopically at 100-200 × magnification. For wound healing assays, 4.9 × 10^4^ cells in 70 µl of medium were seeded into Culture-Insert (Ibidi). After the cells were confluent, to inhibit the effect of cell proliferation, the cells were pretreated with 10 µg/ml mitomycin C (Sigma) for 2 h, and washed with culture medium. After removal of Culture-Insert, cells were incubated with fresh media and photographs of the migration assay were taken at 0, 5, 15, 20, 25 and 30 h using a phase-contrast microscope with digital camera. The cell migration was quantified by calculating the cell-covered area using WimScratch software (Wimasis).

### Proliferation assay

The cells were placed in a 6-well plate at a concentration of 3 × 10^4^ cells per well. After incubation for 1 to 4 days, cells were trypsinized and resuspended in 3 ml of appropriate medium. Cell suspensions were centrifuged at 1000 rpm for 5 min. Cell pellets were resuspended in 1 ml of appropriate medium. The viable cells were counted with a hemocytometer after trypan blue staining.

### Cell viability detection

Cell viability was determined using the cell proliferation reagent MTS (Promega) following the manufacturer's instruction.

### Statistical analysis

We performed statistical analysis using the unipolar, paired Student *t*-test. The significance of the data was accepted when the *P* value was less than 0.05.

### Funding

This work was supported by grant from Basic Science Research Program through the National Research Foundation of Korea (NRF) funded by the Ministry of Education, Science and Technology (NRF-2011-0010805), and National R&D Program for Cancer Control, Ministry of Health, Welfare and Family affairs, Republic of Korea (0820050).

### CONFLICT OF INTEREST

Authors have no conflict of interests

## SUPPLEMENTARY FIGURES AND TABLES


