# Supplementary material for: RhoGDI2 promotes epithelial-mesenchymal transition via induction of Snail in gastric cancer cells
Source: Oncotarget. 2014 Feb 7;5(6):1554–64. doi: 10.18632/oncotarget.1733 (PMC4039231; doi:10.18632/oncotarget.1733)
Supplement: Supplementary file 1 [file oncotarget-05-1554-s001.pdf]

RhoGDI2 promotes epithelial-mesenchymal transition via induction of Snail in gastric cancer cells – Cho et al

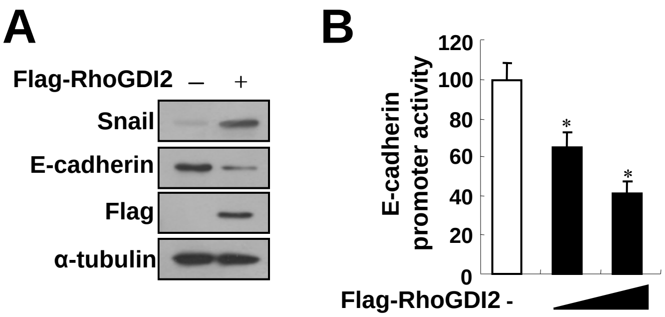

Supplementary Figure 1. RhoGDI2 increases Snail expression and decreases E-cadherin expression. (A) HEK293T cells were transiently transfected with RhoGDI2-expressing plasmid (Flag-RhoGDI2) or control plasmid (pCMVTag2B) and immunoblotted with indicated antibodies. (B) Effect of RhoGDI2 on E-cadherin promoter activity in HEK293T cells. Data are mean ± SD of three individual experiments, each in triplicate. ∗, $P < 0.01$ as determined by paired Student *t* test.

Supplementary Table 1

Supplementary Table 1. Primer sequences used for RT-PCR analyses

Name of Gene	Direction	Nucleotide sequences
E-cadherin	Sense	5’-CGAGTCCCCTAGTCGTCCT-3’
	Antisense	5’-TGATTCTGCTGCTCTTGCTG-3’
Snail	Sense	5’-TGCAGTATTTGCAGTTGAAG-3’
	Antisense	5’-CAGAGTTTACCTTCCAGCAG-3’
Twist	Sense	5’-AGCTTGCCATCTTGGAGT-3’
	Antisense	5’-TACGAGGAGCTGCAGACG-3’
Slug	Sense	5’-CCCCAAAGATGAGGAGTATC-3’
	Antisense	5’-CAAACTACAGCGAACTGGAC-3’
β-actin	Sense	5’-CCAGATCATGTTTGAGACCT-3’
	Antisense	5’-TTGAAGGTAGTTTCGTGGAT-3’
